# Clinical study on the application of biological mesh and synthetic mesh in laparoscopic inguinal hernia repair

**DOI:** 10.1007/s00423-026-03990-y

**Published:** 2026-02-17

**Authors:** Shuai Chang, Yao Zhao, Di Zhang, Shunle Li, Hongjun Zhai, Hong Ji

**Affiliations:** Department of General Surgery, The Second Affiliated Hospital of Xi’an Jiaotong University, Xi’an, 710004 People’s Republic of China

**Keywords:** Inguinal hernia, Laparoscopic, Synthetic mesh, Acellular matrix mesh, Complications​

## Abstract

**Objective:**

To evaluate the efficacy and safety of biological mesh in laparoscopic inguinal hernia repair.

**Methods:**

A retrospective analysis was conducted on 200 patients who underwent laparoscopic transabdominal preperitoneal repair (TAPP) between March 2019 and March 2022. Patients were divided into Group A (*n* = 100), repaired with acellular matrix biological mesh, and Group B (*n* = 100), repaired with polypropylene mesh. Follow-ups were performed at 1, 3, 6 months, 1 year, and 3 years postoperatively. Outcome measures included operation time, intraoperative blood loss, postoperative hospital stay, recurrence, infection, seroma, foreign body sensation, and chronic pain.

**Results:**

No significant differences were observed between the two groups in operation time, intraoperative blood loss, postoperative hospital stay, recurrence, infection, seroma, or chronic pain (*P* > 0.05). However, the incidence of foreign body sensation was significantly lower in the Group A (*P* < 0.05).

**Conclusion:**

The acellular matrix biological mesh is safe and effective in the treatment of inguinal hernia, and can reduce the incidence of postoperative foreign body sensation at the same time, providing a new choice for laparoscopic inguinal hernia repair.

## Introduction

Inguinal hernia is a common surgical disease. With the development of treatment strategies, reducing the use of permanent implanted meshes and avoiding the long-term effects of meshes on the body are the current development trends. More than 30 years ago, Lichtenstein reported the tension-free repair of inguinal hernia [1], which has since become the most widely used surgical procedure in the treatment of inguinal hernia [2]. As for the repair materials, polypropylene synthetic mesh is the most widely used in the tension-free repair of inguinal hernia at present. Its use has greatly reduced the recurrence rate after inguinal hernia surgery. However, its related complications, such as acute and chronic mesh infection, chronic pain, foreign body sensation in the inguinal region, erosion of adjacent organs, etc., have attracted more and more attention [3]. Biological mesh has become a new research hotspot in the field of hernia surgery due to its good tissue compatibility, anti-infective property and degradability [4]. The acellular tissue matrix of porcine small intestinal submucosa (SIS) mesh is a new type of hernia repair material. At present, there are few literature reports on its repair effect, especially the effectiveness of it in the common surgical procedure TAPP for inguinal hernia has not been verified by high-quality randomized controlled clinical studies. Therefore, this study compared the clinical data of the application of acellular matrix biological mesh and polypropylene mesh in TAPP to verify the safety and effectiveness of the acellular matrix biological mesh, and provide a basis for the application of biological mesh in laparoscopic inguinal hernia repair.

## Materials and methods

### Study population and design

This study was reviewed by the Medical Ethics Committee of the Second Affiliated Hospital of Xi’an Jiaotong University, and informed consent was obtained from all patients. This retrospective comparative study enrolled patients consecutively between March 2019 and March 2022 in the Second Affiliated Hospital of Xi’an Jiaotong University. Patients were allocated to the Group A (biological mesh) or Group B (polypropylene mesh) based on chronological order of admission, without randomization, reflecting real-world clinical practice. Among them, 100 patients were in the Group A, using the acellular matrix biological mesh of porcine small intestinal submucosa (15 × 10 cm) produced by Shaanxi Bio Regenerative Medicine Co., Ltd., and the other 100 patients were in the Group B, using the lightweight large-mesh 3D polypropylene mesh (Shanshi 15 × 9 cm) produced by Beijing Transeasy Medical Technology Co., Ltd., and all underwent TAPP surgery. Inclusion criteria: (1) meeting the diagnostic criteria of inguinal hernia, confirmed by B-ultrasound examination; (2) age ≥ 18 years old; (3) non-emergency surgery. Exclusion criteria: (1) recurrent hernia, bilateral hernia or incarcerated hernia; (2) history of abdominal surgery; (3) combined with malignant tumor, immune system diseases, potential infection; (4) combined with severe liver and kidney insufficiency; (5) coagulation dysfunction.

### Surgical technique

**Fig. 1:**
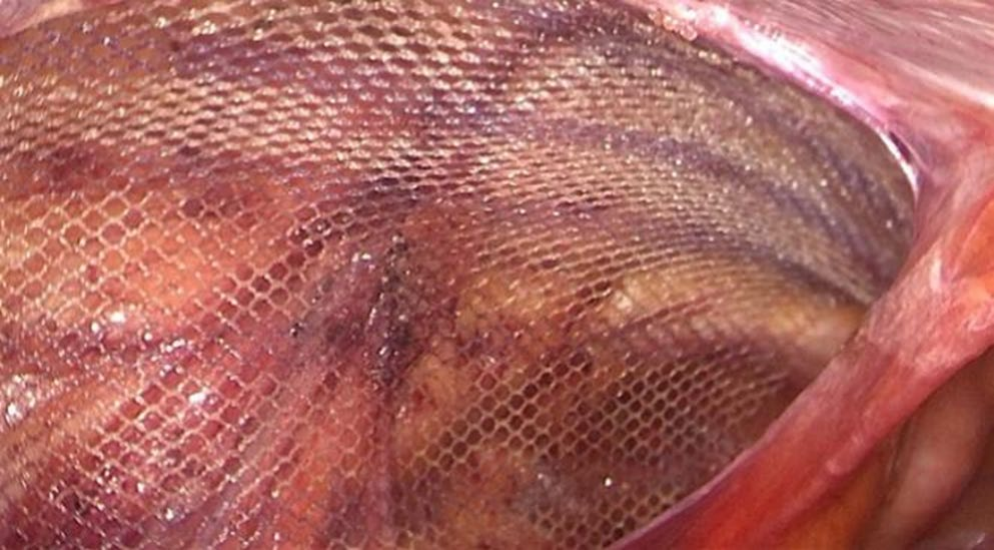
Placement of polypropylene patch

**Fig. 2:**
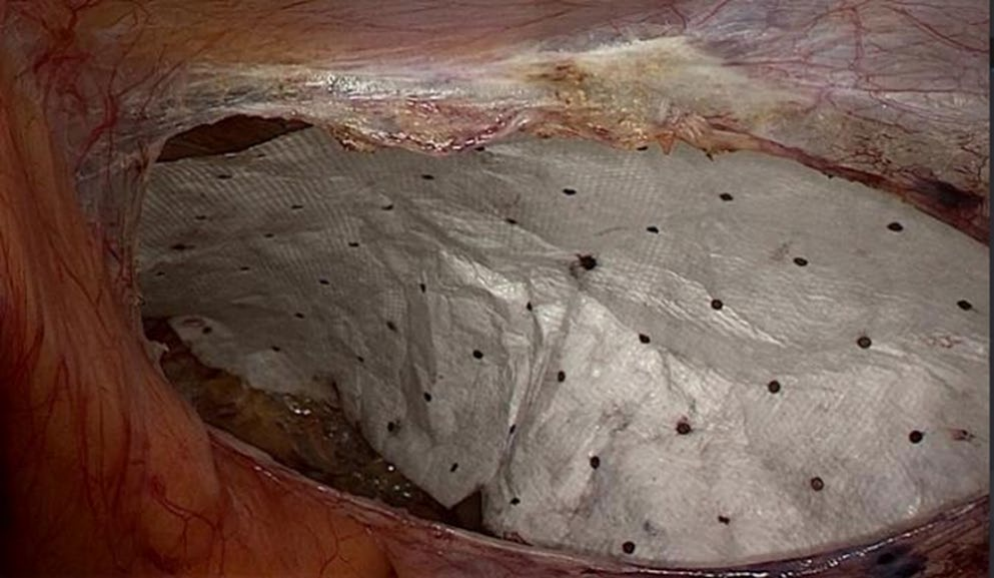
Placement of acellular matrix biological patch

TAPP procedure: All surgeries were performed by a skilled surgeon. Combined intravenous-inhalation general anesthesia was used, and the patient was placed in a head-low and foot-high position at an angle of 15°. A urinary catheter was inserted before the operation. Surgical process: (1) Placement of trocars: The pneumoperitoneum pressure was 12 mmHg. A 10 mm trocar was inserted above the umbilicus as an observation port, and a 30° laparoscope was inserted. Under direct vision, a 5 mm trocar was inserted on each side of the rectus abdominis muscle beside the umbilicus as an operating port; (2) Separation of the preperitoneal space: The peritoneum was incised 2 cm above the hernia ring, extending outward to the anterior superior iliac spine and inward not exceeding the medial umbilical fold. The preperitoneal space was dissected to expose the inferior epigastric vessels, pubic symphysis, iliopubic tract, pectineal ligament, etc. The hernia sac was dissected and pulled back, and the dissection was continued until the spermatic cord (or round ligament of the uterus) was denuded of the peritoneum. During the process, attention was paid to avoiding damage to the blood vessels; (3) Placement and fixation of the mesh: The mesh was placed into the abdominal cavity through the umbilical observation port and placed into the dissected preperitoneal space. The mesh completely covered the myopectineal orifice area. The mesh was fixed at 4–5 points with medical glue (n-butyl cyanoacrylate), including the pectineal ligament, conjoint tendon, etc. (Figs. 1 and 2); (4) Suture and closure of the peritoneum: The peritoneal fissure was continuously sutured with 3 − 0 absorbable suture or barbed suture to close the peritoneum.

### Observation index

The operation time, intraoperative blood loss, and postoperative hospital stay of the two groups were recorded respectively. The patients were followed up in the outpatient department at 1, 3, 6 months, 1 year, and 3 years after the operation. The recurrence rate, the infection rate of the incision and the mesh, seroma, and foreign body sensation were evaluated by physical examination and pelvic CT examination, which was performed only if physical examination suggested recurrence or complications (e.g., unexplained pain, palpable mass). The visual analogue scale (VAS) was used to evaluate the postoperative pain of the patients in the two groups. Postoperative pain lasting longer than 3 months with a VAS score of ≥ 3 is defined as chronic pain. The assessment of foreign body sensation was conducted using the Carolinas Comfort Scale (CCS) [5, 6].

### Statistical analysis

SPSS 24.0 statistical software was used to analyze the data. Count data were expressed as cases or percentages (%), and chi-square (*χ*^2^) test was performed; measurement data were expressed as mean ± standard deviation, and t-test was performed for comparison between the two groups. Repeated variance test was used for comparison at different time points. A p-value of < 0.05 was considered statistically significant.

## Results

The patients in both the Group A and the Group B completed the surgery smoothly and the postoperative follow-up was also completed smoothly. There were no significant differences in general data such as age, gender, body mass index, and type of inguinal hernia between the two groups of patients (Table 1). There were no significant differences in operation time, intraoperative blood loss, and postoperative hospital stay (Table 2). Within 3 years, there was no recurrence in both groups, and there was no infection of the incision and the mesh. There were no significant differences in the incidence of seroma and chronic pain between the two groups. The incidence of foreign body sensation after operation in the Group A was significantly lower than that in the Group B (*P* < 0.05), and the difference was statistically significant (Table 3).

**Table 1:** Comparison of general data between the two groups

Variables	Gender		Age (years)	BMI	Type of hernia		
	Male	Female		(kg/m^2^)	Indirect	Direct	Femoral
Group B(*n* = 100)	84	16	60.8 ± 7.8	23.39 ± 1.75	64	32	4
Group A(*n* = 100)	88	12	58.8 ± 7.6	22.96 ± 1.64	67	28	5
*P*-value	0.67		0.20	0.21	0.80		

**Table 2:** Comparison of surgical situations between the two groups

Variables	Operation time /min	Intraoperative blood loss /ml	Postoperative hospital stay /d
Group B(*n* = 100)	60.46 ± 9.72	6.58 ± 2.32	1.15 ± 0.14
Group A(*n* = 100)	61.42 ± 7.78	6.06 ± 2.31	1.18 ± 0.18
*P*-value	0.58	0.28	0.81

**Table 3:** Comparison of the incidence of complications between the two groups

Variables	Recurrence	Infection	Seroma	Chronic pain	Foreign body sensation
Group B(*n* = 100)	0	0	8	3	10
Group A(*n* = 100)	0	0	10	2	2
*P*-value			0.62	1.00	0.03

## Discussion

For adult inguinal hernia, tension-free hernia repair is a recognized and effective surgical procedure. With the development of laparoscopic technology, laparoscopic tension-free inguinal hernia repair, including laparoscopic transabdominal preperitoneal inguinal hernia repair (TAPP) and laparoscopic totally extraperitoneal inguinal hernia repair (TEP), has become the main surgical procedure for inguinal hernia, and the proportion is increasing rapidly [7, 8]. Compared with the traditional open surgical method, laparoscopic surgery has the advantages of less pain, quick recovery, and low wound infection rate [9, 10]; in terms of the repair range, laparoscopic surgery can cover the entire myopectineal orifice. Compared with traditional surgery, theoretically, it can reduce the recurrence rate, and for the situation where there are two or more types of hernias on the same side at the same time, it can reduce the missed diagnosis rate. With the increasing application of laparoscopic surgery, the situation of contralateral occult hernia is gradually increasing in clinical practice. Bilateral hernia repair can be completed without increasing the number of puncture holes, which is another major advantage of laparoscopic surgery [11]. It is precisely because of these advantages that more and more hospitals and doctors are more inclined to choose laparoscopic surgery for patients who can tolerate it.

At present, the main repair material for inguinal hernia repair is still the synthetic mesh, and the polypropylene mesh is the most common. Most artificial synthetic meshes strengthen the abdominal wall or repair the defect by inducing the host’s foreign body reaction and generating dense scar tissue that wraps the mesh. While artificial synthetic meshes reduce the risk of postoperative recurrence, they also bring some complications, such as mesh infection, enteric fistula or bladder fistula caused by mesh erosion, local foreign body sensation, chronic pain [12]; due to the formation of local inflammatory reaction and scar tissue, the compliance of the abdominal wall will be reduced. For women who have not given birth, the synthetic mesh is not suitable. For unmarried and childless men, the possible vas deferens obstruction caused by the synthetic mesh should be considered. Although many reports believe that the impact of the synthetic mesh on fertility is unclear, the use of synthetic mesh for unmarried and childless men should be cautious [13, 14].

The acellular tissue matrix mesh is the most widely used type of biological mesh at present. It is derived from animal tissues. The acellular technology is used to remove various cells contained in the tissues, and the extracellular matrix containing collagen and the three-dimensional framework are retained. The mechanism of tissue repair after implantation into the human body is “endogenous tissue regeneration”: (1) After implantation, the matrix is rapidly revascularized, and circulating stem cells enter the damaged tissue; (2) Stem cells accumulate and adhere to the matrix in this area; (3) Gradually differentiate into specific tissue cells; (4) The newly differentiated cells regenerate new matrix for the regenerated tissue. This repair process will not form a large amount of scar tissue, and there is no permanent foreign body remaining in the body. Theoretically, it avoids the related complications caused by the inflammatory reaction, such as mesh infection, erosion, etc. The newly formed tissue has the same physiological function and strength as the healthy tissue. It should be noted that precisely because a biologic mesh is eventually absorbed by the body, its long-term recurrence risk remains controversial. Based on the above characteristics, biological mesh is the main research direction of the next generation of hernia repair materials [15, 16].

There are many types of biological meshes, among which porcine-derived materials and bovine-derived materials are the most common. The acellular tissue matrix material of porcine small intestinal submucosa has obvious advantages compared with materials such as acellular dermal matrix and collagen sponge: (1) It has good hydrophilicity, which is conducive to the infiltration of body fluids into the material and vascularization, and is conducive to the growth of cells into the interior of the material, promoting tissue repair; (2) It has high mechanical strength and can meet the needs of abdominal wall repair. A small number of foreign literature reports suggest that biological mesh can be applied in open hernia repair or laparoscopic hernia repair [17, 18]. In recent years, with the development of domestic biological meshes and the decrease in price, the proportion of biological mesh application in inguinal hernia repair has gradually increased, and its actual effect needs to be proved by more clinical trials to provide data support for the clinical application of biological mesh.

This study showed that the use of biological mesh in TAPP is safe and effective. During the 3-year follow-up period after the operation, compared with the synthetic mesh, there were no significant differences in the recurrence rate, seroma, mesh infection rate, chronic pain, etc., and the incidence of local foreign body sensation was lower, which is consistent with the degradable property of the biological mesh. As for the possible local erosion caused by the synthetic mesh, such as erosion of the intestine or bladder, leading to enteric fistula or bladder fistula, theoretically, it is impossible to occur with the biological mesh.

Biological mesh can be well integrated into the body, providing a three-dimensional scaffold, and degrading after the growth of its own tissue. It has the required tensile strength and anti-infective ability, and has a very good application prospect. In the old view, biological mesh was only considered for special populations (such as those who have not given birth, patients with incarcerated hernia). Following centralized procurement, the price of domestically produced biological mesh has become comparable to or only slightly higher than that of polypropylene mesh. In this study, biological mesh costs merely CNY 2,000 more than polypropylene mesh, which remains well within patients’ affordability thresholds. As price differentials diminish, the adoption of biological mesh in hernia repair for the general population is progressively increasing.

In conclusion, the use of biological mesh in TAPP is safe and feasible. Without increasing the operation time and recurrence risk, the incidence of local foreign body sensation is lower. We will continue the follow-up observation in the future and further expand the sample size to further observe and summarize the long-term curative effect of the biological mesh.

## Data Availability

The datasets used and/or analyzed during the current study are available from the corresponding author on reasonable request.
